# Timing of Term Births and Associated Mortality Risks: Ecological Analysis Across 28 European Countries

**DOI:** 10.1111/1471-0528.18292

**Published:** 2025-07-10

**Authors:** Jóhanna Gunnarsdóttir, Marianne Philibert, Mika Gissler, Karin Källén, Kari Klungsøyr, Marzia Loghi, Alison Macfarlane, Luule Sakkeus, Vlad Tica, Jennifer Zeitlin

**Affiliations:** ^1^ Faculty of Medicine University of Iceland Reykjavik Iceland; ^2^ Department of Obstetrics and Gynecology Landspitali—The National University Hospital of Iceland Reykjavik Iceland; ^3^ Obstetrical Perinatal and Pediatric Epidemiology Université Paris Cité, Inserm Paris France; ^4^ Department of Data and Analytics THL Finnish Institute for Health and Welfare Helsinki Finland; ^5^ Department of Molecular Medicine and Surgery Karolinska Institutet Stockholm Sweden; ^6^ The National Board of Health and Welfare, Department of Evaluation and Analysis Epidemiology and Methodological Support Unit Stockholm Sweden; ^7^ Department of Global Public Health and Primary Care University of Bergen Bergen Norway; ^8^ Division of Mental and Physical Health The Norwegian Institute of Public Health Bergen Norway; ^9^ Directorate for Social Statistics and Welfare Italian Statistic Institute—Istat Rome Italy; ^10^ Centre for Maternal and Child Health Research City St George's, University of London London UK; ^11^ Estonian Institute for Population Studies Tallinn University Tallinn Estonia; ^12^ University “Ovidius”, County University Emergency Hospital Constanta Romania; ^13^ Academy of Romanian Scientists Bucharest Romania

**Keywords:** early‐term birth, neonatal death, perinatal death, stillbirth

## Abstract

**Objective:**

To explore term mortality rates in relation to rates of early‐term birth (gestational ages 37 + 0 to 38 + 6 weeks), regarded as a proxy indicator of practices of elective birth by induction or caesarean.

**Design:**

Ecological study using national birth data.

**Setting:**

28 European countries.

**Population:**

Births ≥ 37 weeks between 2015 and 2020.

**Methods:**

Aggregated data on live and stillbirths by completed week of gestation was compiled from routine sources in the Euro‐Peristat network. Countries were divided into three groups based on their percentages of early‐term births using terciles (high, medium and low) and mortality rates were compared between groups with random‐effects meta‐analysis of proportions.

**Main Outcome Measures:**

Stillbirths (antepartum or intrapartum fetal death) and perinatal death (stillbirth or early neonatal death) per 1000 total births ≥ 37 weeks.

**Results:**

Early‐term birth rates ranged from 17.8% (Iceland) to 49.1% (Cyprus), with terciles being < 21%, 21%–27%, and > 27%. Post‐term birth rates were low in countries with higher early‐term birth rates. The pooled stillbirth rate ≥ 37 weeks was 1.28 per 1000 total births (95% CI: 1.13–1.46) in the lowest tercile and 1.05 (95% CI: 0.95–1.16) in the highest (*p* = 0.05), but prediction intervals were wide reflecting heterogeneity within groups. No evidence of difference was seen between perinatal mortality rates by tercile (*p* = 0.71).

**Conclusion:**

On average, the stillbirth rate was lower in countries where early‐term birth rates were highest, but no difference was found in perinatal mortality rates. Heterogeneity was high within groups.

## 1 Introduction

Stillbirths can occur unexpectedly in pregnancies with no previous signs of complications. Attempts are increasingly made to prevent stillbirth in term pregnancy by inducing labour to shorten the time at risk in utero [1, 2, 3]. Unfortunately, the lack of accurate screening methods to detect pregnancies at risk of stillbirth makes it difficult to target the use of induction to prevent stillbirths [4, 5]. Induction is therefore being offered to large groups of women with only moderate risks of stillbirth. These include women with uncomplicated pregnancies that have reached 41 gestational weeks (late term) [6, 7, 8, 9, 10] and pregnancies at 39–40 weeks and 6 days (full term) in women over 39 years of age, regardless of other risk factors [11]. Although most stillbirths cannot be anticipated, some pregnancy complications such as pre‐eclampsia [12], fetal growth restriction and pre‐gestational diabetes are strongly associated with stillbirth [13]. In some countries, induction at early term (37–38 weeks and 6 days) is recommended in pregnancies complicated by these conditions, and in some cases caesarean section may be indicated. Over the past 50 years, the stillbirth rate in pregnancies affected by pre‐eclampsia has decreased substantially [14, 15]. This is because the condition has increasingly been diagnosed in time to intervene with birth before the event of fetal death.

There is a lack of international consensus about acceptable indications for planned early‐term births. Recently, an increasing proportion of early‐term labour inductions have been justified by pregnancy complications that have limited association with stillbirth, such as mild cholestasis [16] and gestational diabetes mellitus [13, 17, 18]. Importantly, the risk of neonatal mortality [19, 20, 21, 22] and morbidity after early‐term births is higher than after births at 39 full weeks of pregnancy [23]. Therefore, most guidelines suggest that births by elective induction or caesarean in uncomplicated pregnancies should not take place before 39 weeks [24, 25]. Despite these recommendations, planned early‐term births without any recorded medical indication have been reported [2, 26, 27]. Further, wide differences have been found between gestational age distributions in European countries, suggesting differences in practices of indicated early‐term births [28], and there is limited evidence about the potential impact of early‐term birth on mortality.

In this ecological study, we aimed to describe stillbirth, neonatal and perinatal death rates in 28 European countries and assess their association with the early‐term birth rate, interpreted as a proxy indicator of practices of elective birth by induction or caesarean.

## 2 Methods

### 2.1 Study Design

This study employs an ecological design using aggregated national‐level birth data from European countries.

### 2.2 Data Sources

National routine birth data was compiled by the Euro‐Peristat network, a collaboration between European countries to compare perinatal health indicators [29]. Euro‐Peristat has reported national‐level statistics on selected perinatal indicators at regular intervals to inform clinical care and policy for pregnant women and babies since its inception in 2000 [30]. Data come from routine sources, including vital statistics, birth registers and hospital discharge data [31]. The network includes epidemiologists, statisticians and clinicians who have expertise in investigating maternal and newborn health [32].

The data used for this study were compiled as part of the European PHIRI (Population Health Information Research Infrastructure), that aimed to share data and expertise on the COVID‐19 pandemic via a population health information portal. In the PHIRI project, a new data collection protocol using a federated framework was conceived and implemented, involving the definition of a common data model [33]. Each participating data provider built a database following the specifications of the common data model and ran R scripts provided by the project coordinator on their server to produce aggregate data tables that were transferred to the central coordination office (see Appendix S1 for contributing partners and data sources for each country). Neither the pregnant population nor the public were involved in planning this research.

### 2.3 Study Population

The study population included all term births at 37 weeks of gestation or more, excluding terminations of pregnancy, from 2015 to 2020 in 28 European countries.

### 2.4 Variables and Definitions

The main outcomes were national‐level stillbirth (antenatal and intrapartum deaths), perinatal death (stillbirths and early neonatal deaths before 7 days after live birth) and neonatal death (death before 28 days after live birth) rates at term. Rates were calculated per 1000 total births at ≥ 37 weeks for the stillbirth and perinatal mortality rates, and per 1000 total live births at ≥ 37 weeks for the neonatal mortality rates.

Our exposure variable was the gestational age distribution at term and, specifically, the early term birth rate, computed as the percentage of term births between 37 + 0 and 38 + 6 weeks of gestational age. Gestational age was defined as the best obstetrical estimate and collected according to completed weeks of gestation.

The caesarean birth rate among term births was also included in this analysis as a covariable. The purpose was to assess if higher rates of early‐term births were associated with more obstetrical intervention. Data were not available on prelabour caesarean or induction of labour at term gestations.

### 2.5 Missing Data

Some countries could not provide data on all outcomes. Poland contributed to the stillbirth analysis for 2018–2020 as data on stillbirths was missing for the period 2015–2017. France, Germany, Italy, Luxembourg, Portugal and Slovakia could not provide data on neonatal mortality because they did not have information about gestational age at birth for neonatal deaths. Ireland reported only early neonatal mortality (< 7 days).

### 2.6 Statistical Analysis

We described the gestational age distribution by comparing the proportions of the following categories of births between countries: early term (37 + 0 to 38 + 6), full term (39 + 0 to 40 + 6), late term (41 + 0 to 41 + 6) and post‐term (42 + 0 or more). Then we divided countries into three groups based on their proportions of early‐term births (High, Medium and Low), using terciles of the distribution to define cut‐offs. Using random‐effects meta‐analysis of proportions, we first compared the caesarean section rates in each group. Then, using the same approach, we derived the pooled estimates of stillbirth, perinatal mortality and neonatal mortality rates for each group. A generalised linear mixed model with a logit transformation was used for the analysis of proportions, as recommended by Schwarzer et al. [34] To evaluate heterogeneity, we calculated the between‐study variance or *τ* ^2^, as recommended for meta‐analysis [35], using the restricted maximum likelihood method with 95% prediction intervals [36]. This measure illustrates the predicted range of the true effect size expected for 95% of similar future studies and is more suited than the *I* ^2^ to our analysis which seeks to explain differences in the effect size [37].

The principal models were run on all countries with data available for a given outcome, but sensitivity analyses were carried out on the set of countries with all outcomes (stillbirth and neonatal mortality) available.

Analyses were performed using R version 4.1.1 (The R Foundation for Statistical Computing, Vienna, Austria) with the ‘meta’ package version (version 5.2‐0).

## 3 Results

**FIGURE 1:**
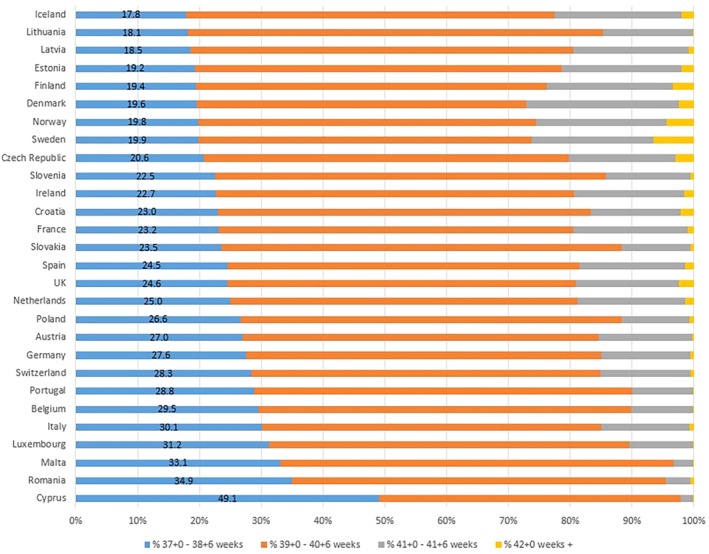
Gestational age distribution of all births ≥ 37 weeks in European countries.

The distribution of gestational age at birth varied markedly between participating countries, as shown in Figure 1 which presents countries ordered by the proportion of early‐term births (with the number of births each week by country presented in Table S1). The early‐term birth rate ranged from 17.8% (Iceland) to 49.1% (Cyprus) and the tercile thresholds to define groups were < 21%, 21%–27% and > 27%. In the tercile with the lowest proportion of early‐term births, the mode of the gestational age of live births was 40 weeks, while it was 39 weeks in the highest tercile (Figure S1).

Figure 1 also shows the post‐term birth rate which varied from 0.1% (Malta, Luxembourg, Portugal, Cyprus) to 6.6% (Sweden). In the tercile with the highest early‐term births (> 27%), the rate of post‐term births was extremely low, ranging between 0.1% and 0.7%, while in the tercile with low early‐term births (< 21%), the rate of post‐term births was considerably higher in some but not in all countries (ranging from 0.2% to 6.6%).

There was wide variation between countries in the caesarean birth rate, ranging from 14.8% (Norway) to 51.9% (Cyprus). The caesarean birth rate was related to the tercile of early‐term birth rate (*p* < 0.001). The pooled caesarean section rate at term was 18.2% in the lowest tercile (95% confidence interval [CI]: 16.5%–20.1%, prediction interval [PI]: 12.4% to 25.9%), compared with 23.5% in the middle tercile (95% CI: 20.1%–27.2%, PI: 12.8%–38.9%) and 31.5% in the highest tercile (95% CI: 24.4%–39.4%, PI: 11.0%–63.0%) (Figure S2).

**FIGURE 2:**
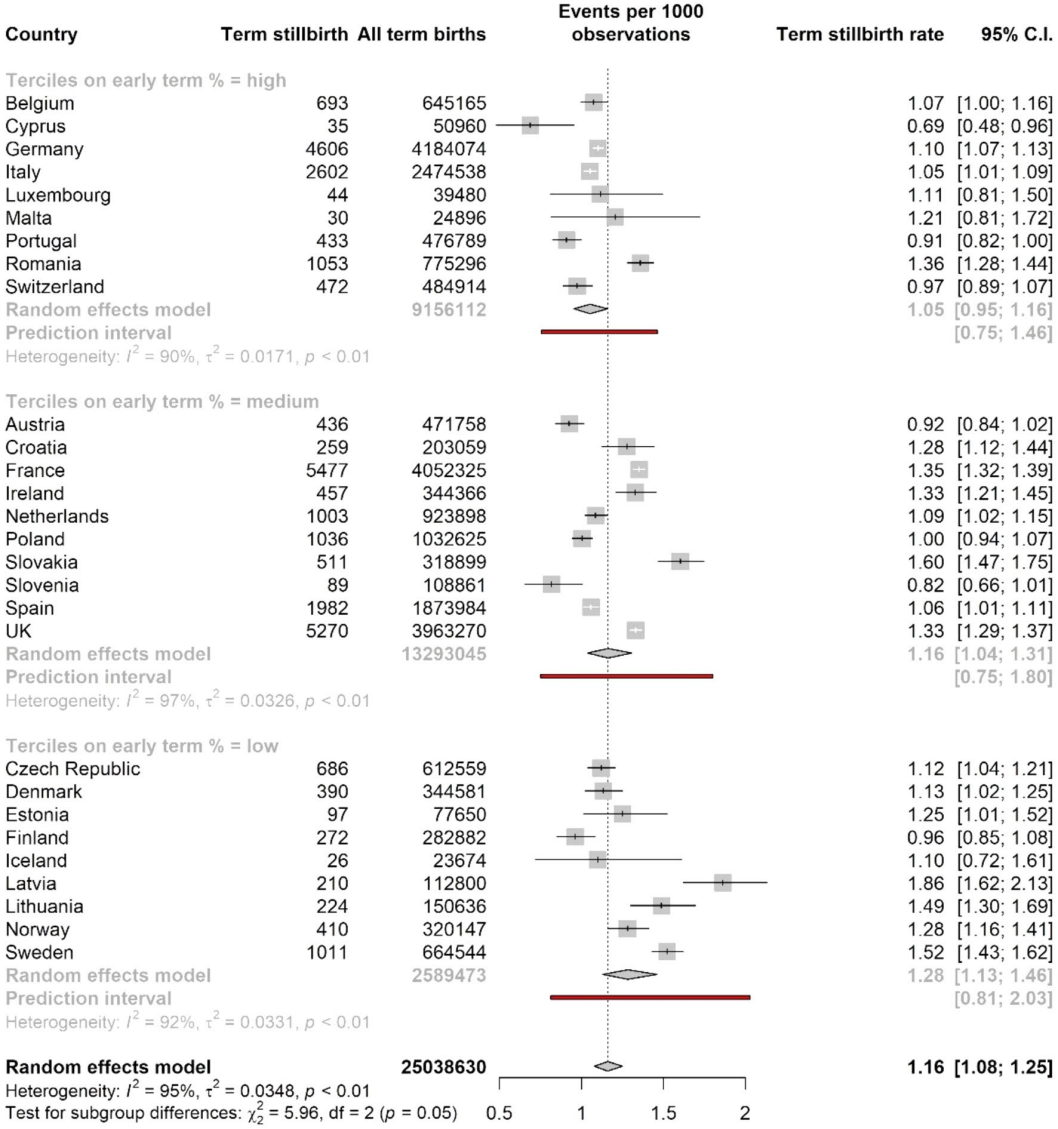
Pooled stillbirth rate at ≥ 37 weeks by tercile of early‐term group, obtained by random‐effects meta‐analysis of proportions.

**FIGURE 3:**
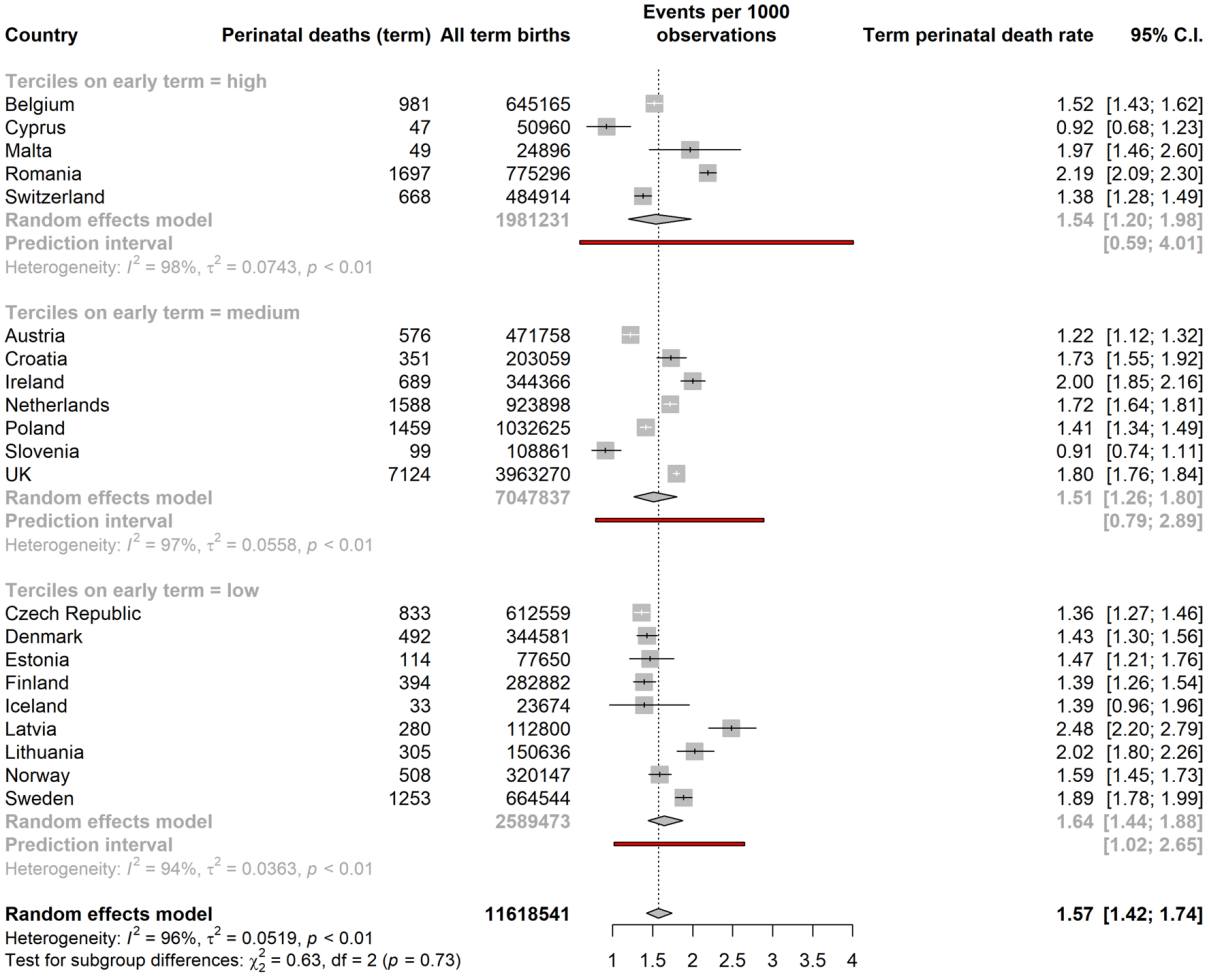
Pooled perinatal death rate at ≥ 37 weeks by tercile of early‐term group, obtained by random‐effects meta‐analysis of proportions.

**FIGURE 4:**
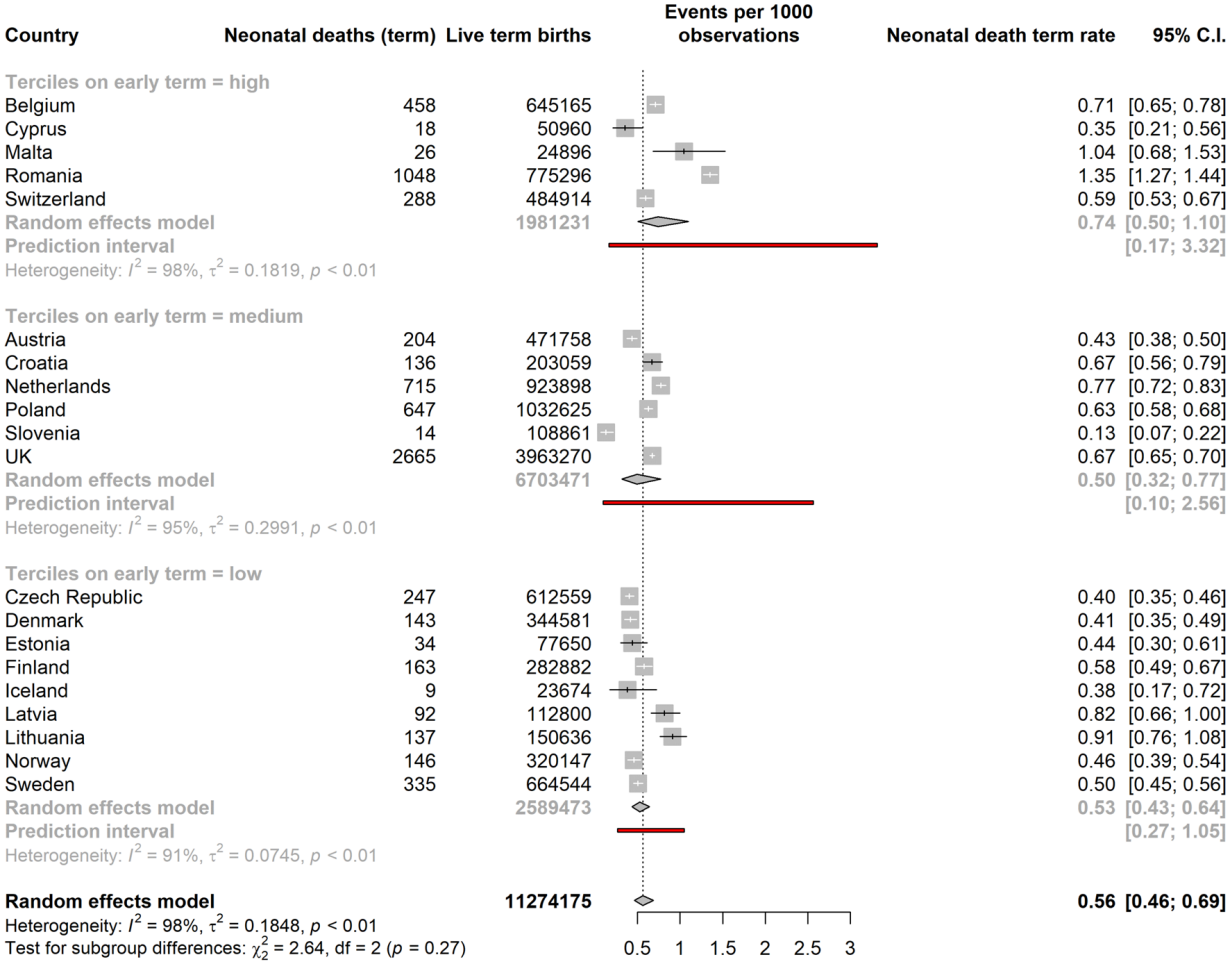
Pooled neonatal death rate at ≥ 37 weeks by tercile of early‐term group, obtained by random‐effects meta‐analysis of proportions.

The stillbirth rate per 1000 total births at ≥ 37 weeks ranged from 0.69 (Cyprus) to 1.52 (Sweden). The pooled rate for all countries from the mixed‐effects meta‐analysis of proportions was 1.16 (95% CI: 1.08–1.25). In subgroup analyses, the pooled estimates for stillbirth rates were highest in lower early‐term birth tertiles: 1.28 per 1000 total births (95% CI: 1.13–1.46; PI: 0.81–2.03) compared to 1.16 (95% CI: 1.04–1.31, PI: 0.75–1.80) in the middle tercile and 1.05 (95% CI: 0.95–1.16, PI 0.75–1.46) in the highest tercile (Figure 2). Prediction intervals were wider than the CI for these pooled estimates, reflecting heterogeneity in mortality rates within tercile groups. Estimates of the pooled perinatal mortality rate at ≥ 37 weeks were similar by tercile: 1.64 per 1000 total births (95% CI: 1.44–1.88, PI: 1.02–2.65) in the lowest tercile, compared with 1.51 (95% CI: 1.26–1.80, PI: 0.79–2.89) in the middle tercile and 1.54 (95% CI: 1.20–1.98, PI: 0.59–4.01) in the highest tercile (Figure 3). For neonatal mortality, the pooled estimates were very close in the lowest and middle terciles, 0.53 (95% CI: 0.43–0.64, PI: 0.27–1.05) and 0.50 (95% CI: 0.32–0.77, PI: 0.10–2.56), respectively, with a higher pooled point estimate of neonatal mortality in the highest tercile of early‐term birth: 0.74 per 1000 live births (95% CI: 0.50–1.10, PI: 0.17–3.32) (Figure 4). Information about the number of neonatal deaths within 7 days and 8–28 days from birth at 37 weeks or more can be found in Table S2. In sensitivity analyses of stillbirth rates, including only the same countries as for perinatal and neonatal deaths, results were almost identical (Figure S3).

## 4 Discussion

### 4.1 Main Findings

This study presents an overview of mortality rates for births at ≥ 37 weeks in relation to gestational age of term pregnancies in European countries. In some countries, early‐term births were common with about one in three births before 39 weeks, compared with fewer than one in five births in other countries. Caesarean section rates were higher in countries where early‐term births were common. Just over one in 1000 total births at ≥ 37 weeks were stillbirths with 0.6 neonatal deaths per 1000 live births. When the pooled rates were compared between countries with the highest and lowest terciles of early‐term births, they differed by about two stillbirths per 10 000 births. There was no evidence of differences in the rates of perinatal or neonatal mortality.

### 4.2 Strengths and Limitations

The strength of this study was the inclusion of national population‐based birth data from 28 European countries. Data were formatted to a validated common data model to allow the production of comparable perinatal indicators, unavailable in other international databases. Further, having data over a 6‐year period provided good precision for term mortality, even in smaller countries. The study's major limitation is its ecological design. Ecological studies are usually placed at the bottom of the evidence pyramid because of the difficulty of adjusting for individual covariables and their vulnerability to bias [38]. In our study, these limits relate specifically to an inability to control for individual risk factors in the childbearing populations or healthcare system factors which could affect early‐term births, caesareans and mortality. Nonetheless, ecological studies play a role in the evidential toolbox by providing important descriptive benchmarks, broadening the settings in which exposure‐outcome associations are explored, and generating hypotheses about patterns that cannot be observed in one country alone. Other limitations of our study relate to missing neonatal death data in some countries, although excluding them in sensitivity analysis did not affect our results. We also lacked other data items, including induction and prelabour caesarean section at term, which were not collected. Ongoing practice changes show the importance of including these indicators in Euro‐Peristat's future work, in addition to integrating adjustments for individual‐level risk factors.

### 4.3 Interpretation

In this study, the stillbirth rate at ≥ 37 weeks was slightly lower in countries in the highest tercile of early‐term births. This was not surprising as the risk of stillbirth has been shown to increase with gestational age from 37 weeks, especially in pregnancies reaching late term and post‐term [39]. However, no difference was found in perinatal mortality in this study. Previous studies have reported higher rates of neonatal death after birth at 37–38 weeks compared with full term [19, 39]. In this study, no difference in neonatal mortality was detected between early‐term birth groups, due in part to high heterogeneity of mortality rates within groups. Observational data suggest that planned early‐term births in pregnancies complicated by severe conditions like pre‐eclampsia could prevent stillbirths without increasing neonatal mortality [14, 15], but no randomised trials on early‐term induction have been powered to detect improved neonatal outcomes. One large trial compared induction at 39 weeks with expectant management in nulliparous low‐risk women in the United States [40], and a similar trial is ongoing in France [41]. In the trial from the United States, a composite neonatal outcome was not significantly different between groups [40]. High‐quality evidence supports late‐term induction rather than expectant management until 42 weeks, but over 400 inductions may be needed to prevent one death [6, 7, 8, 9, 10, 39]. Although firm conclusions cannot be drawn from these results due to the ecologic design, one possible explanation for the small differences seen in this study between stillbirth rates in terciles of early‐term birth could be the number of pregnancies that reached late term rather than a direct impact of planned early‐term births on stillbirth.

Growing evidence suggests that increasing the early‐term birth rate may negatively affect not only neonatal mortality [19] but also morbidity in infancy and childhood such as neonatal respiratory distress, chronic lung diseases in infancy and hospital admissions, especially due to infections [20, 21, 22]. There are also reports suggesting negative effects of early‐term birth on the cognitive function of infants and children [42, 43, 44], whereas planned full‐term births may not affect childhood developmental outcomes adversely [45]. Therefore, it is important to appreciate that the wide differences between countries in gestational age distributions at ≥ 37 weeks can potentially affect neonatal and child health beyond the scope of this study. This is important as the terciles of early‐term birth may reflect differences in rates of elective births by caesarean or induction. Indeed, we found the highest overall caesarean birth rates in countries with the highest early‐term birth rates. The wide range of caesarean birth rates from 14% to 46% contrasts with the recommendation by The European Association of Perinatal Medicine and the European Midwives Association of a country‐level caesarean birth rate of 15%–20% [46]. Higher rates do not reduce mortality rates but can increase complications including placenta previa spectrum disorder in later pregnancies [47] and uterine rupture during attempted vaginal births after a previous caesarean [46]. Countries with the lowest caesarean birth rates, such as Norway with a caesarean birth rate at 15%–16% since 2001, have maintained a stable rate for at least two decades. In contrast, European countries with the highest rates followed the rise observed in USA from 1996 to 2006 [48]. This was accompanied by a sharp increase in the percentage of early‐term birth in the USA from 19% in 1992 and peaking at 31% in 2006. In 2009, guidelines were issued in the USA recommending against non‐medically indicated caesareans before 39 weeks [49]. In 2013, the ‘Term pregnancy work group’ proposed the terminology used in this paper to categorise births at ≥ 37 weeks into early‐term, full‐term, late‐term and post‐term births [50]. Despite these recommendations, the increase in early‐term births in USA has been only partially reversed [51]. The impact of the rising rates of labour induction in many European countries on the gestational age distribution should also be considered. For example, rates of early‐term induction have been rising since 2006 in Denmark, Iceland and Finland [2, 51]. Importantly, there has been no recorded clinical indication for some of these early‐term inductions [2]. International recommendations are therefore urgently needed to guide the appropriate use of early‐term labour induction, with justified clinical indications giving details of benefit and harm. A conservative use of interventions within reason would be preferable to fast changes in clinical practice, as changes can quickly become the new ‘norm’ making them hard to reverse even if new data suggest a harmful effect.

## 5 Conclusion

The stillbirth rate was lower in countries where early‐term birth rates were highest, but there was high heterogeneity within groups. No difference was found in the perinatal or neonatal mortality rates. These results should be interpreted with caution, as differences in mortality rates can be affected by many factors related to the overall quality of care, in addition to gestational age at birth. Importantly, there were wide differences between gestational age distributions within Europe, and in countries with high early‐term birth rates, the caesarean section rate was high. Therefore, variation in early‐term birth may reflect broader systemic differences in obstetric practice, and policy caution is warranted before extrapolating population‐level associations to individual care decisions. Stakeholders should be aware of the large differences in gestational age distribution within Europe, because children born at early term may have worse long‐term health outcomes than children born at full term.

## Author Contributions

All authors were involved in the conception of this study and planning analysis. M.P. carried out the analysis under the supervision of J.Z. J.G. and M.P. wrote the first draft of the paper in close collaboration. All authors critically reviewed the manuscript and accepted the final version that was submitted.

## Ethics Statement

Data used for the study are aggregated at the country level and therefore, ethics committee approvals are not required, in accordance with European data regulations governing anonymised data.

## Conflicts of Interest

The authors declare no conflicts of interest.

## Supporting information


**Appendix S1.** Data sources and data providers of Euro‐Peristat participating in the PHIRI protocol.
**Table S1.** Number of births by weeks of gestation at ≥ 37 weeks in 2015–2020, ordered by percentage of early term births.
**Table S2.** Number of neonatal deaths after live birth at ≥ 37 weeks by timing of death, ordered by percentage of early term births.
**Figure S1.** Distribution of gestational age of births ≥ 37 weeks, by early‐term group.
**Figure S2.** Pooled caesarean rate at ≥ 37 weeks by tercile of early‐term group, obtained by random‐effects meta‐analysis of proportions.
**Figure S3.** Pooled stillbirth rate at ≥ 37 weeks by tercile of early‐term group, including only the same countries as the analysis for perinatal death.

## Data Availability

The data that support the findings of this study are available from the corresponding author upon reasonable request.
