# Supplementary material for: Timing of Term Births and Associated Mortality Risks: Ecological Analysis Across 28 European Countries
Source: BJOG. 2025 Jul 10;132(11):1655–63. doi: 10.1111/1471-0528.18292 (PMC12411659; doi:10.1111/1471-0528.18292)

**Appendix A: Data sources and data providers of Euro-Peristat participating in the PHIRI protocol**

|  | **Data sources** | **Data providers** |
| --- | --- | --- |
| **Austria** | * Birth statistics (Statistics Austria)  * Cause of death statistics (Statistics Austria) | * Statistics Austria |
| **Belgium** | * Vital Statistics, Statistics Belgium (Statbel) | * Statbel |
| **Croatia** | * Croatian Medical Birth Database (Croatian Public Health Institute),  * Croatian Mortality Database (Croatian Central Bureau of Statistics) - | * Croatian Institute of Public Health |
| **Cyprus** | * Medical Birth register (Health Monitoring Unit, Cyprus Ministry of Health)  * Causes of Death register (Health Monitoring Unit, Cyprus Ministry of Health)  * Database for COVID-19 confirmed cases and deaths (Health Monitoring Unit, Cyprus  Ministry of Health) | * Health Monitoring Unit, Ministry of Health |
| **Czech Republic** | * Institute of Health Statistics and Information of the Czech Republic (national birth register  (mothers and newborns) collecting individual perinatal data.) | * Institute of Health Information and Statistics of the  Czech Republic |
| **Denmark** | * Medical birth register (The Danish Data authority, Danish Ministry of Health)  * National patient register (The Danish Data authority, Danish Ministry of Health)  * Danish causes of death register (The Danish Data authority, Danish Ministry of Health)  * The Centralized Civil Register | * Statistics Denmark |
| **Estonia** | * Estonian Medical Birth Register (National Institute for Public Health) was linked with data from  * Estonian Cause of Death Register (National Institute for Public Health) | * Estonian Institute for Population Studies, Tallinn  University |
| **Finland** | * Medical Birth Register (Finnish Institute for Health Welfare) linked with Central Population  Register (Digital and Population Data Services Agency) and Cause of Death Register  (Statistics Finland)  * Register on Induced Abortions (Finnish Institute for Health Welfare) for late terminations  22–24 weeks | * Finnish Institute for Health and Welfare (THL) |
| **France** | * Hospital discharge data ( *Programme de Médicalisation des Systèmes d'Information* (PMSI))  in the French National System of Health Data ( *Système national des données de santé (*SDNS)) | * Department for Research, Studies, Assessment  and Statistics (DREES), French Ministry of Health |
| **Germany** | * IQTIG (Federal Institute for the Quality of Medical Care)  * Destatis (Federal Statistical Office) | * IQTIG |
| **Iceland** | * The Icelandic Birth Registration  * Hospital register (National University Hospital) | * National University Hospital |
| **Ireland** | *National Perinatal Reporting System (the Healthcare Pricing Office) | * Healthcare Pricing Office |
| **Italy** | * Birth certificates (Ministry of Health)  * Causes of deaths (Istat)  * Terminations of pregnancies (Istat)  * Miscarriages (Istat) | * Italian National Institute for Statistics-ISTAT |
| **Latvia** | * Newborn Register of Latvia (Centre for Disease Prevention and Control of Latvia)  * Register of Causes of Death (Centre for Disease Prevention and Control of Latvia) | * The Centre for Disease Prevention and Control of  Latvia |
| **Lithuania** | * Medical Date of Births (Institute of Hygiene Health Information Centre)  * Database of the Demographic Statistics (Central Statistical Office)  * Causes of Death register (Institute of Hygiene Health Information Centre) | * Institute of Hygiene, Health Information Centre |
| **Luxembourg** | * Perinatal Health Monitoring System (Luxembourg Institute of Health)  * National Causes of Death Registry (Directorate of Health of Luxembourg) | * Department of Population Health, Luxembourg  Institute of Health  * Directorate of Health of Luxembourg |
| **Malta** | * National Obstetrics Information System (Directorate for Health Information and Research)  * National Mortality Register (Directorate for Health Information and Research) | * Directorate for Health Information and Research |
| **Netherlands** | * Perined (The Netherlands Perinatal Registry) | * Perined |
| **Norway** | * Medical Birth Register of Norway (The Norwegian Institute of Public Health) | * The Norwegian Institute of Public Health |
| **Poland** | * Central Statistical Office  * Ministry of Health | * National Research Institute of Mother and Child |
| **Portugal** | * Instituto Nacional de Estatística – Portugal (Statistics Portugal)  * Central Administration of the Health System | * Institute of Public Health of the University of Porto |
| **Romania** | * National Institute for Public Health Romania | * National Institute of Public Health Romania |
| **Slovakia** | *National Health Information Center | * National Health Information Center |
| **Slovenia** | *Perinatal information system (National institute of public health) | * University Medical Centre, Research Unit |
| **Spain** | * Vital Statistics (National Statistics Office)  * Specialized Care Registry - Minimum Basic Data Set (Ministry of Health) | * Senior Statistical State Corps and Public Health and  Addictions Directorate, Generalitat Valenciana |
| **Sweden** | * Medical Birth Register (The National Board of Health and Welfare) | * The National Board of Health and Welfare |
| **Switzerland** | * Vital Statistics (BEVNAT) | * Swiss Federal Statistical Office |
| **UK, all** | * MBRRACE UK (University of Oxford and University of Leicester) | * University of Leicester, MBRRACE-UK collaboration |
| **UK, England, and Wales** | *UK, Office for National Statistics (Live birth and stillbirth registration in England and Wales,  birth notification in England and Wales) | * Office for National Statistics |
| **UK, Northern Ireland** | * Northern Ireland Maternity System - NIMATS | * Public Health Agency (Northern Ireland) |
| **UK, Scotland** | * Scottish Morbidity Record 02 (maternity hospital discharge record)  * National Records of Scotland Stillbirth, live birth, and infant death registrations (statutory  vital event registration) | *Public Health Scotland |
| **UK, Wales** | *Digital Health and Care *Wales* | *Digital Health and Care *Wales* ( *DHCW*) |

**Appendix B:**

**Table S1:** Number of births by weeks of gestation at ≥ 37 weeks in 2015-2020,
ordered by percentage of early term births

| **COUNTRY** | **37 weeks** | **38 weeks** | **39 weeks** | **40 weeks** | **41 weeks** | **≥42 weeks** | **All births  ≥37 weeks** |
| --- | --- | --- | --- | --- | --- | --- | --- |
| Iceland | 1439 | 2782 | 6571 | 7559 | 4 854 | 469 | 23 674 |
| Lithuania | 7935 | 19329 | 41687 | 59476 | 21 985 | 224 | 150 636 |
| Latvia | 6228 | 14670 | 30275 | 39595 | 21 115 | 917 | 112 800 |
| Estonia | 4709 | 10195 | 21255 | 24854 | 15 067 | 1 570 | 77 650 |
| Finland | 15060 | 39708 | 76947 | 83735 | 57 929 | 9 503 | 282 882 |
| Denmark | 19391 | 48000 | 78386 | 105218 | 85 432 | 8 154 | 344 581 |
| Norway | 19447 | 43995 | 78263 | 96496 | 67 973 | 13 973 | 320 147 |
| Sweden | 37847 | 94037 | 165637 | 192850 | 130 505 | 43 668 | 664 544 |
| Czech Republic | 37107 | 89305 | 165409 | 196437 | 106 034 | 18 267 | 612 559 |
| Slovenia | 7499 | 17028 | 32945 | 35936 | 14 913 | 540 | 108 861 |
| Ireland | 24014 | 54082 | 98665 | 100853 | 61 215 | 5 537 | 344 366 |
| Croatia | 13906 | 32841 | 58317 | 64008 | 29 633 | 4 354 | 203 059 |
| France | 272846 | 666001 | 1187450 | 1134871 | 753 446 | 37 711 | 4 052 325 |
| Slovakia | 20900 | 54160 | 85931 | 120542 | 35 366 | 2 000 | 318 899 |
| Spain | 150540 | 309043 | 502510 | 564162 | 321 927 | 25 802 | 1 873 984 |
| UK | 352591 | 620679 | 1143885 | 1086416 | 666 468 | 93 231 | 3 963 270 |
| Netherlands | 73610 | 157194 | 249167 | 270563 | 160 656 | 12 708 | 923 898 |
| Poland* | 75223 | 207180 | 336001 | 300236 | 108 501 | 5 484 | 1 032 625 |
| Austria | 35033 | 92104 | 131252 | 141018 | 70 858 | 1 493 | 471 758 |
| Germany | 334328 | 820088 | 1154354 | 1246108 | 605 277 | 23 919 | 4 184 074 |
| Switzerland | 36162 | 101235 | 132901 | 141425 | 70 277 | 2 914 | 484 914 |
| Portugal | 40207 | 96974 | 164280 | 127828 | 46 827 | 673 | 476 789 |
| Belgium | 55369 | 135151 | 200273 | 189352 | 63 957 | 1 063 | 645 165 |
| Italy | 198775 | 546970 | 704919 | 651843 | 353 832 | 18 199 | 2 474 538 |
| Luxembourg | 3586 | 8732 | 11542 | 11534 | 4 036 | 50 | 39 480 |
| Malta | 2142 | 6101 | 7984 | 7873 | 771 | 25 | 24 896 |
| Romania | 61981 | 208850 | 282015 | 187292 | 30 892 | 4 266 | 775 296 |
| Cyprus | 7512 | 17513 | 14266 | 10573 | 1 025 | 71 | 50 960 |

*Births in 2018-2020, because Poland has no registration of stillbirths in 2015-2017.

**Table S2:** Number of neonatal deaths after live birth at ≥ 37 weeks by timing of death, ordered by percentage of early term births.

|  | **Number of neonatal deaths** | | |
| --- | --- | --- | --- |
| **COUNTRY** | **Early**  **0-7 days** | **Late**  **8-28 days** | **Timing**  **unknown** |
| Iceland | <10 | <10 | NA |
| Lithuania | 81 | 56 | NA |
| Latvia | 70 | 22 | NA |
| Estonia | 17 | 17 | NA |
| Finland | 122 | 41 | NA |
| Denmark | 102 | 41 | NA |
| Norway | 98 | 48 | NA |
| Sweden | 242 | 93 | NA |
| Czech Republic | 147 | 100 | NA |
| Slovenia | 10 | <10 | NA |
| Ireland | 232 | NA | NA |
| Croatia | 92 | 44 | NA |
| France | NA | NA | NA |
| Slovakia | NA | NA | NA |
| Spain | NA | NA | NA |
| UK | 1854 | 811 | NA |
| Netherlands | 585 | 109 | 21 |
| Poland* | 423 | 224 | NA |
| Austria | 140 | 64 | NA |
| Germany | NA | NA | NA |
| Switzerland | 196 | 92 | NA |
| Portugal | NA | NA | NA |
| Belgium | 288 | 170 | NA |
| Italy | NA | NA | NA |
| Luxembourg | NA | NA | NA |
| Malta | 19 | <10 | NA |
| Romania | 644 | 390 | 14 |
| Cyprus | 12 | <10 | NA |

*Births in 2018-2020, because Poland has no registration of stillbirths in 2015-2017.

#N/A: Not applicable or data not available.

**Figure S1**: Distribution of gestational age of births ≥ 37 weeks, by early-term group

**Figure S2:** Pooled caesarean rate at ≥ 37 weeks by tercile of early-term group, obtained by random-effects meta-analysis of proportions.
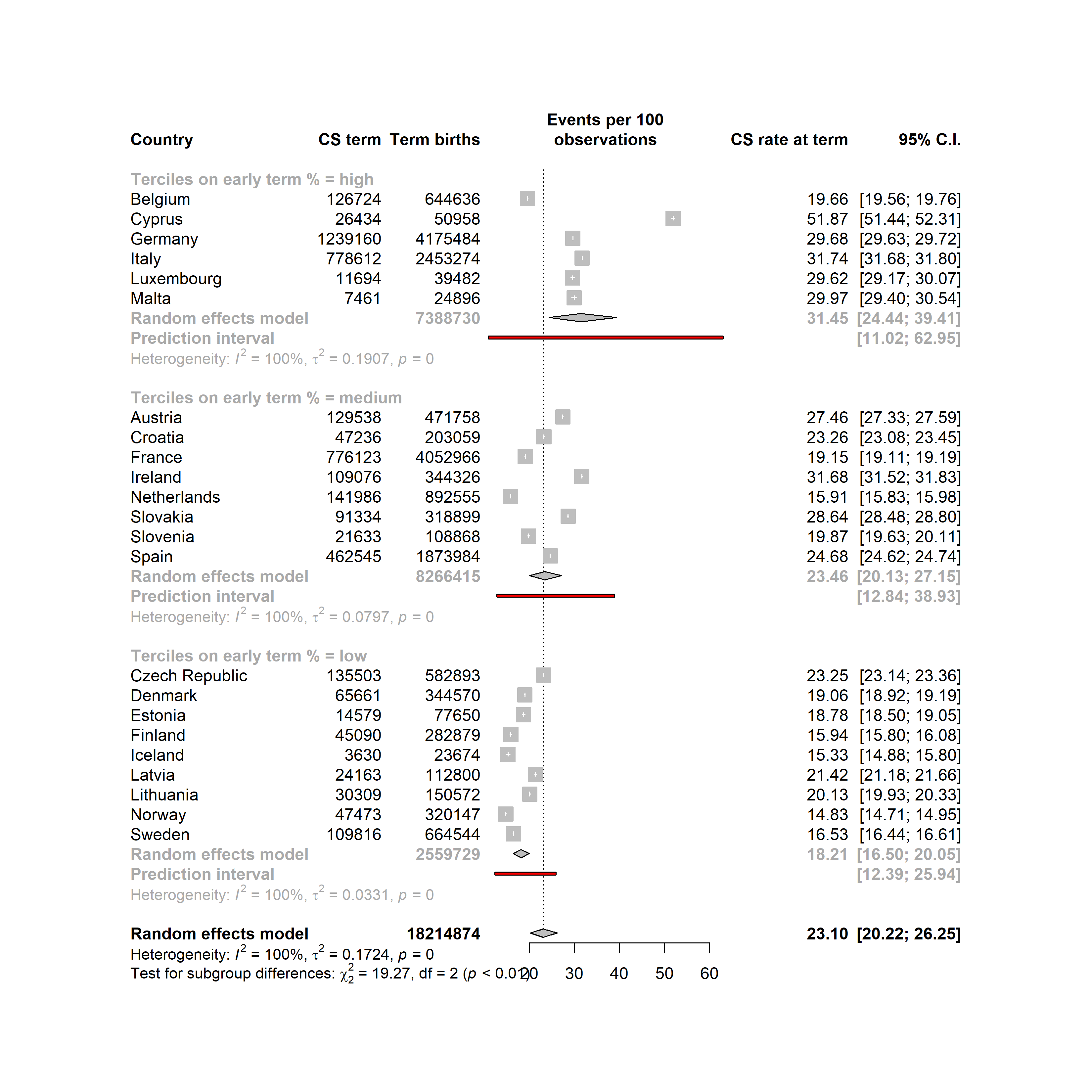


**Figure S3:** Pooled stillbirth rate at ≥ 37 weeks by tercile of early-term group, including only the same countries as the analysis for perinatal death.


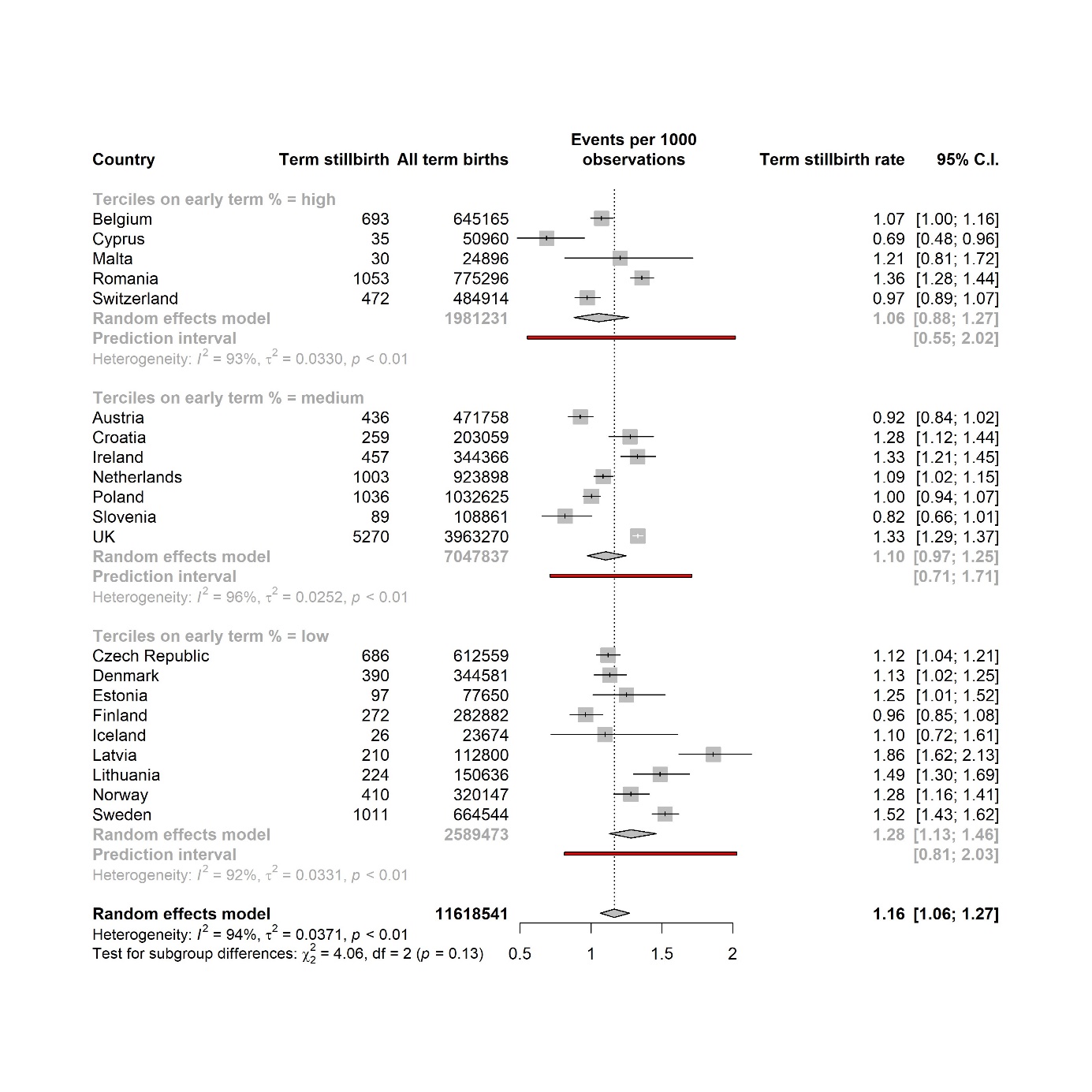

Supplement: Supplementary file 1 — Appendix S1. Data sources and data providers of Euro‐Peristat participating in the PHIRI protocol. Table S1. Number of births by weeks of gestation at ≥ 37 weeks in 2015–2020, ordered by percentage of early term births. Table S2. Number of neonatal deaths after live birth at ≥ 37 weeks by timing of death, ordered by percentage of early term births. Figure S1. Distribution of gestational age of births ≥ 37 weeks, by early‐term group. Figure S2. Pooled caesarean rate at ≥ 37 weeks by tercile of early‐term group, obtained by random‐effects meta‐analysis of proportions. Figure S3. Pooled stillbirth rate at ≥ 37 weeks by tercile of early‐term group, including only the same countries as the analysis for perinatal death. [file BJO-132-1655-s001.docx]
